# Full-scale performances of the slab track subgrade filled with basalt fiber-reinforced foamed concrete

**DOI:** 10.1007/s40534-024-00363-3

**Published:** 2025-01-10

**Authors:** Zhichao Huang, Qian Su, Wenhui Zhao, Zongyu Zhang, Junjie Huang, Sakdirat Kaewunruen

**Affiliations:** 1 School of Civil Engineering, Southwest Jiaotong University, Chengdu, 610031 China; 2 Department of Civil Engineering, School of Engineering, University of Birmingham, Birmingham, B15 2TT England; 3 Key Laboratory of High-Speed Railway Engineering of Ministry of Education, Southwest Jiaotong University, Chengdu, 610031 China; 4 School of Civil Engineering, Lanzhou Jiaotong University, Lanzhou, 730070 China

**Keywords:** High-speed railway, Slab track subgrade, Basalt fiber-reinforced foamed concrete, Model testing, Dynamic performances

## Abstract

Foamed concrete has been used to address the issue of differential settlement in high-speed railway subgrades in China. However, to enhance crack resistance, reinforcement is still necessary, and further research is required to better understand the performance of foamed concrete in subgrade applications. To this end, a series of tests—including uniaxial compressive and dynamic triaxial tests—were conducted to comprehensively examine the effects of basalt fiber reinforcement on the mechanical properties of foamed concrete with densities of 700 and 1000 kg/m^3^. Additionally, a full-scale model of the foamed concrete subgrade was established, and simulated loading was applied. The diffusion patterns of dynamic stress and dynamic acceleration within the subgrade were explored, leading to the development of experimental formulas to calculate the attenuation coefficients of these two parameters along the depth and width of the subgrade. Furthermore, the dynamic displacement and cumulative settlement were analyzed to evaluate the stability of the subgrade. These findings provide valuable insights for the design and construction of foamed concrete subgrades in high-speed rail systems. The outcomes are currently under consideration for inclusion in the code of practice for high-speed rail restoration.

## Introduction

Slab track system with high stability and low maintenance costs has been widely used in high-speed railways [1–4], particularly in China. By the end of 2023, the mileage of China’s high-speed railways had reached 45,000 km, with 70% of these lines utilizing slab tracks. Numerous studies [5–16] have indicated that this type of composite system is sensitive to subgrade settlement, especially differential and excessive settlement (beyond the adjustment range of fasteners), which can directly affect the smoothness of railway, threatening the safety and comfort of high-speed trains. As a result, settlement control become a critical focus in construction and maintenance of high-speed slab track systems.

**Fig. 1:**
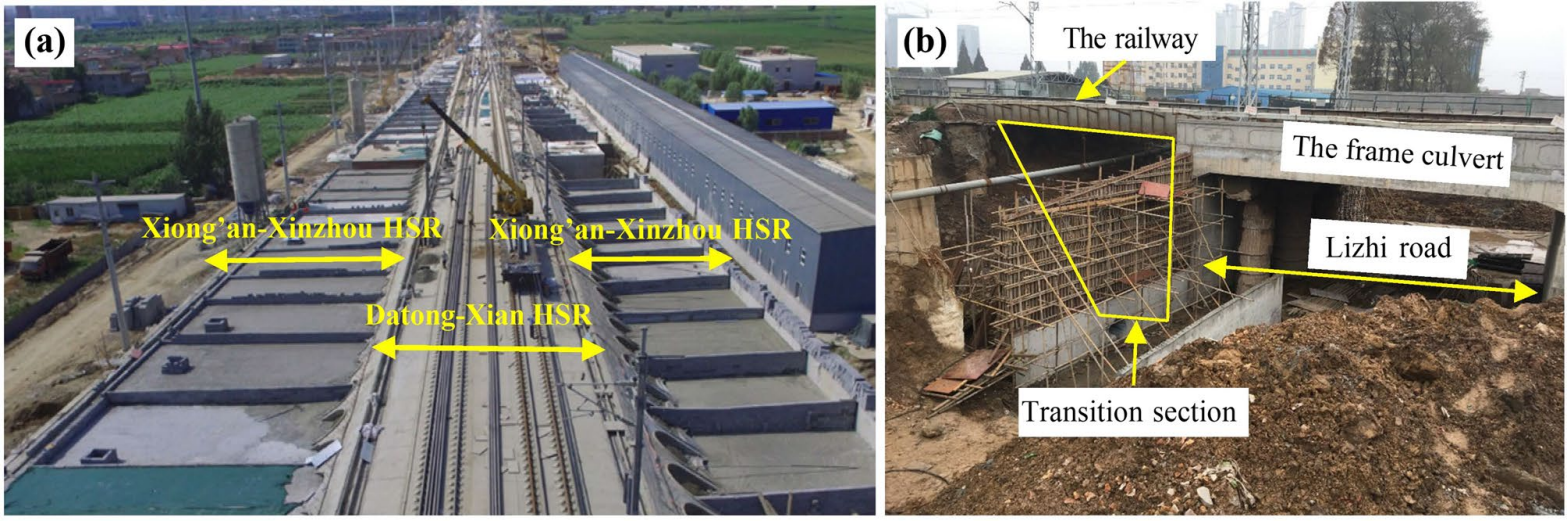
Foamed concrete subgrade: **a** transition section construction in Lizhi Road in Wuhan; **b** subgrade widening of Xinzhou West Railway Station

Foamed concrete, known for its low density, strength, and energy absorption, has garnered attention from researchers and engineers. This material has been employed in high-speed railway subgrades, particularly in widened subgrades and transition zones, to control settlement (as illustrated in Fig. 1). Huang et al. [17, 18] conducted a series of large-model tests to investigate the mechanical behavior of the ballastless track subgrades filled with foamed concrete. Their results showed that after 2 million cycles of loading, the cumulative settlement of the subgrade was less than 1 mm. Additionally, centrifugal model testing and numerical analysis [19] revealed that one year after construction, the settlement at the center of the surface of a widened subgrade filled with foamed concrete was approximately 45% to 56% less than that of a subgrade filled with traditional fillers. Furthermore, a numerical model for a high-speed railway bridge approach [20] indicated that settlement in a subgrade filled with graded crushed stones exceeded that in a foamed concrete-filled subgrade by over 1.5 times. Foamed concrete in the transition section effectively reduced ground settlement by approximately 79% compared to traditional materials. A filed test [21] demonstrated that the maximum settlement in a foamed concrete subgrade-culvert transition zone within the first 200 days of construction was around 6.21 mm, well below the 30 mm limit specified for maintaining smooth high-speed lines [22]. Overall, the use of foamed concrete in subgrades has proven effective in controlling settlement due to its low density.

Several studies [17–21, 23, 24] have identified the optimal density range for foamed concrete in railway subgrade applications, which is between 500 and 1000 kg/m^3^ to meet the requirements of design codes for strength of filling materials [22, 25]. In accordance with the technical specifications for railway foamed concrete subgrade construction [26], the bottom layer of the subgrade bed should have a density of no less than 650 kg/m^3^, and the embankment should have a density of no less than 550 kg/m^3^. However, long-term cyclic loading can induce cracks in foamed concrete-filled subgrade beds [17], potentially compromising stability and safety. Therefore, enhancing the dynamic properties of foamed concrete is essential for ensuring subgrade’s long-term performance. Laboratory tests [27–31] have demonstrated that the incorporating basalt fibers significantly improves the physical and mechanical properties of foamed concrete. Compared to other fibers (such as polyvinyl alcohol, glass, and polypropylene), basalt fiber is the most cost-effectiveness when reinforcing sand, making it a promising material for improving foamed concrete in railway subgrades [32].

Dynamic behaviors of foamed concrete subgrades play a crucial role in both performance assessment and maintenance of high-speed railway subgrade. Several researchers have conducted meaningful studies in this area. Huang et al. [17] performed large-scale model tests on foamed concrete subgrades, analyzing long-term performance under cyclic dynamic loads. Their results indicated that, with a loading frequency of 5 Hz and a loading stress amplitude of 24 kPa, dynamic stress and dynamic acceleration in the subgrade stabilized after 0.8 million cycles. Additionally, both values decreased more rapidly along the subgrade depth compared to traditional subgrades. Another study [18] involving a cyclic loading test of a new slab track subgrade, consisting of a graded crushed stone layer, foamed concrete layer, and foundation, showed that the foamed concrete layer significantly improved the diffusion of dynamic stress and acceleration in the subgrade. Specifically, dynamic stress and acceleration at the bottom of the foamed concrete layer were reduced by approximately 39.8%, and 64.5%, respectively, compared to the subgrade surface. Using numerical simulation methods, Liu et al. [23] analyzed the dynamic performance of the bridge-subgrade transition zone filled with foamed concrete for the high-speed railway. They found that the application of foamed concrete can not only improve the distribution of the dynamic stress and dynamic acceleration along the longitudinal direction of the line, significantly reducing the values, but also enhance the diffusion of dynamic stress and dynamic acceleration along the subgrade depth. Furthermore, field test results [21] on the transition subgrade of a ballasted railway indicated that foamed concrete can facilitate the diffusion of dynamic stress and dynamic acceleration within the subgrade bed. While these studies have provided valuable insights into the application of foamed concrete in railway subgrades, certain issues remain. First, in many previous studies, whether involving slab track systems or ballasted track systems, foamed concrete has generally been used to fill only the area beneath the subgrade bed, with the bed itself still filled with graded crushed stone. However, in current practice, slab track subgrades are fully filled with foamed concrete, including both the subgrade bed and body. The dynamic performance of this fully foamed concrete subgrade requires further investigation. Besides, dynamic mechanical behaviors of the subgrade are influenced by train speed, with dynamic response values varying at different positions within the subgrade—both in terms of depth and transversal location. Thus, exploring these dynamic mechanical behaviors is essential for optimizing the design and maintenance of slab track subgrades filled with foamed concrete.

Min–Max normalization is a simple yet effective method to rescale the values of a dataset (usually numerical) so that they fall within a specific range, typically between 0 and 1. When the range of values across different features varies significantly, direct comparisons or analysis can result in some features dominating the results while others are overlooked. Min–Max normalization scales all features to the same range (typically [0, 1]), ensuring that each feature is treated equally in the analysis. This makes it easier to identify genuine patterns and relationships in the data. Furthermore, some features may have much larger ranges than others, causing analysis to become biased toward these dominant features. Min–Max normalization helps reduce this bias by ensuring that all features contribute equally to the analysis, enabling a more comprehensive understanding of the underlying patterns in the data. Therefore, this method can be used to partly eliminate the influence of train speed and measurement point position on the amplitude of dynamic response indicators in the roadbed.

In this study, a series of uniaxial compressive and dynamic triaxial tests were conducted to evaluate the improvement effects of basalt fiber on the mechanical properties of foamed concrete. A full-scale model of a slab track subgrade filled with basalt fiber-reinforced foamed concrete was then constructed, and extensive measurements of dynamic stress, dynamic acceleration, dynamic displacement, and cumulative settlement were carried out. Using the test data, the influence of loading frequency on the subgrade’s dynamic response was assessed. Additionally, the diffusion patterns of dynamic stress and acceleration within the subgrade were analyzed, and empirical formulas were proposed to describe these patterns. Dynamic displacement and cumulative settlement were also evaluated. The findings of this study provide valuable insights for the design and performance assessment of slab track subgrades filled with foamed concrete.

## Physical model of the subgrade filled with the basalt fiber-reinforced foamed concrete

### Full-scale model of the subgrade

**Fig. 2:**
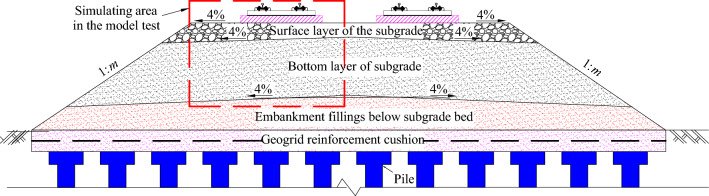
Typical cross section of the slab track subgrade. Here, 1: *m* represents the slope, and the value of *m* is determined by specific design requirements

**Fig. 3:**
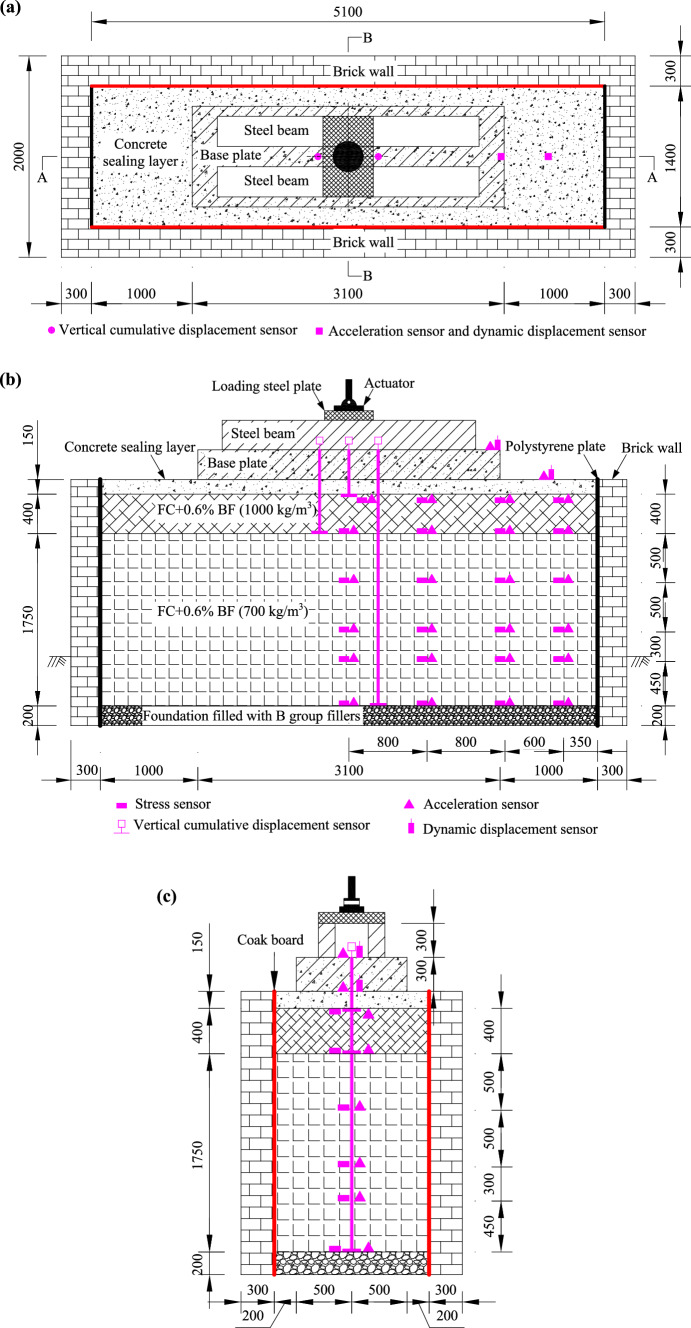
Full-scale section views of the subgrade: **a** plan view; **b** A-A section view; **c** B-B section view (unit: mm)

Figure 2 depicts the typical cross-sectional view of a slab track subgrade. Due to laboratory constraints and in reference to previous research results [33], this paper adopts a simplified model of the subgrade, with the simulated areas highlighted in Fig. 2. A full-scale test track for the slab track subgrade was constructed at Southwest Jiaotong University, with a large brick wall tank measuring 5.1 m × 2.5 m × 1.4 m (width × depth × length), as depicted in Fig. 3. Specifically, a concrete base plate with dimensions 3.1 × 1.0 × 0.3 m was built according to the Chinese high–speed railway design code [25]. Both the surface layer and base layer of the subgrade were filled with foamed concrete, while the foundation layer was filled with group B fillers. To minimize the boundary effect in this model, all four inner surfaces of the brick tank were fitted with energy dissipation layers. As shown in Fig. 3, the two inner short sides of the tank were lined with 10-mm-thick polystyrene plates. Since installing polystyrene l on the longer sides was challenging, cork boards of the same thickness (10 mm) were used instead.

### Properties of the foamed concrete

**Fig. 4:**
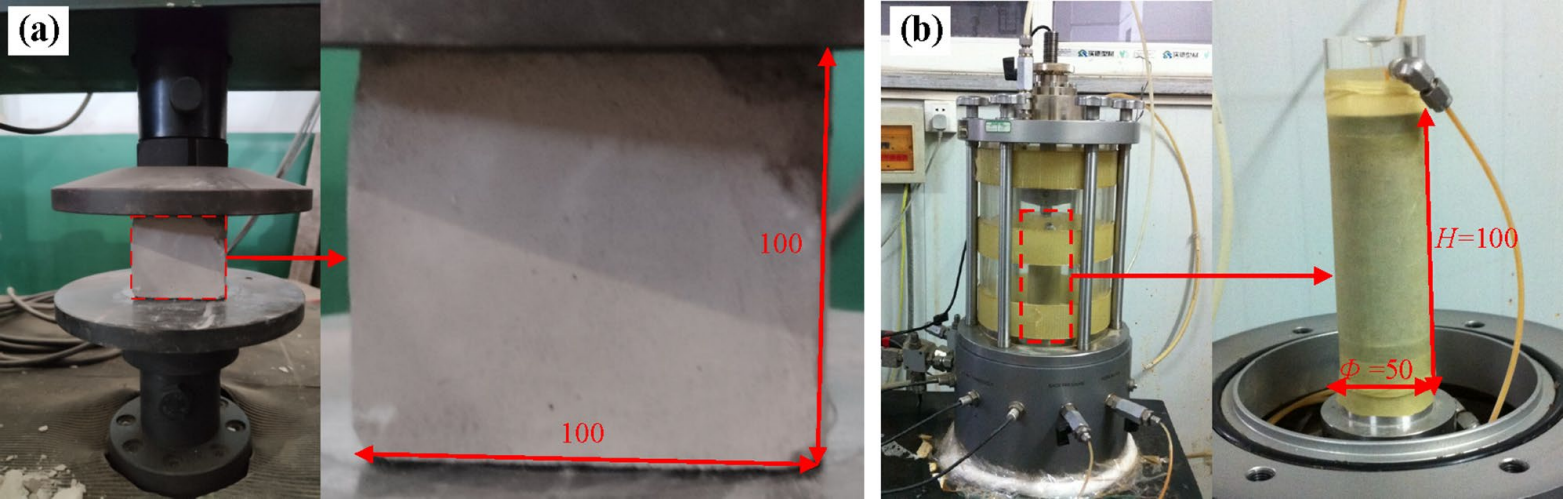
Laboratory tests of the foamed concrete: **a** the uniaxial compressive testing apparatus and sample dimensions; **b** the dynamic triaxial testing apparatus and sample dimensions (unit: mm)

In this model testing, the surface layer of the subgrade bed was filled with foamed concrete at a density of 1000 kg/m^3^ (FC1000), while the underlying subgrade was filled with foamed concrete at a density of 700 kg/m^3^ (FC700). A series of laboratory tests were then conducted to investigate the mechanical properties of the foamed concrete reinforced with basalt fibers. For this study, Portland cement and a protein-based foam agent were used, with a water–cement ratio of 0.65 and a foaming agent to water volume ratio of 0.02. The basalt fiber used in the tests had a diameter of 13 μm and a length of 6 mm. Following the technical specifications for foamed concrete applications [26] and for the soil test of railway engineering [34], uniaxial compressive tests were conducted on samples with two different densities and five fiber content levels. Additionally, dynamic triaxial tests were carried out to compare the dynamic properties of the foamed concrete with and without basalt fiber reinforcement. Figure 4 shows the dimensions of the samples used: 100 × 100 × 100 mm for the uniaxial compressive tests (Fig. 4a) and *Ф* 50 × 100 mm for the dynamic triaxial tests (Fig. 4b). Six distinct levels of basalt fiber content were designed for the study: 0.0%, 0.2%, 0.4%, 0.6%, 0.8%, and 1.0%. For each fiber content level, three samples of foamed concrete were prepared, and the mechanical properties of each were determined by s averaging the test results from the three samples.

**Fig. 5:**
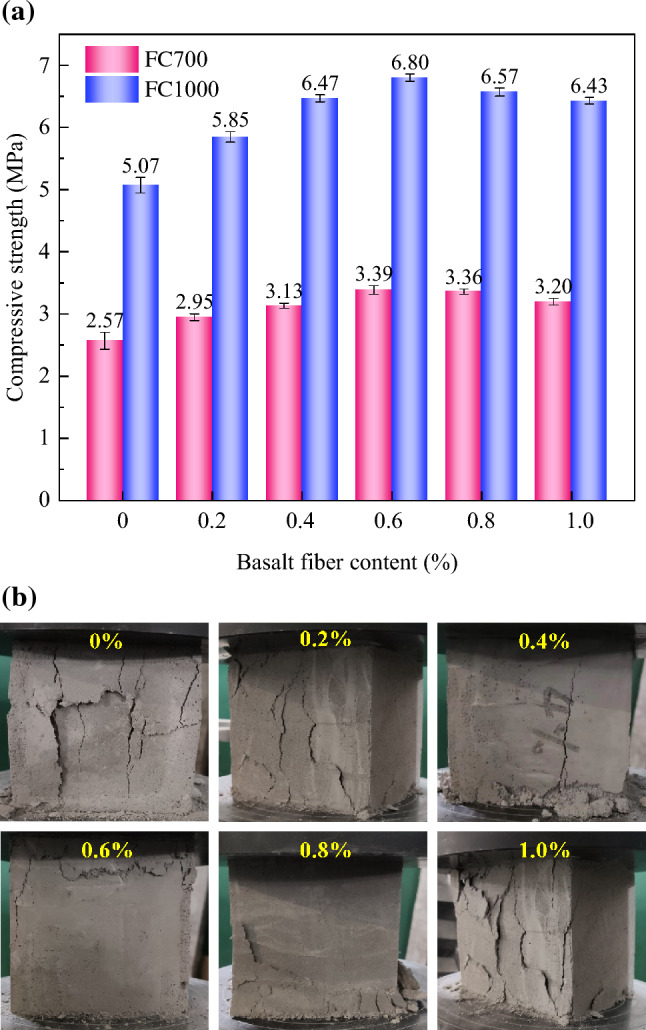
Compressive test results of foamed concrete with different basalt fiber contents: **a** compressive strength; **b** crack patterns in FC700 samples

**Fig. 6:**
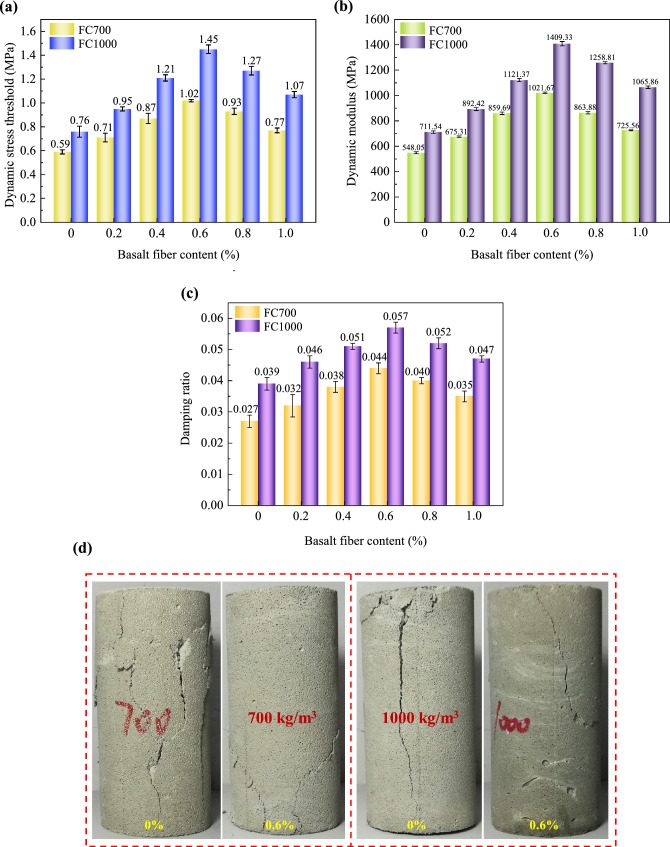
Dynamic triaxial test results of foamed concrete with different basalt fiber contents: **a** dynamic stress threshold; **b** dynamic modulus; **c** damping ratio; **d** crack patterns

As shown in Figs. 5 and 6, an interesting trend emerges as the basalt fiber content increases from 0% to 0.6%. The compressive strengths gradually rise, reaching peak values of 3.39 MPa for the foamed concrete with a density of 700 kg/m^3^ (FC700) and 6.80 MPa for the foamed concrete with a density of 1000 kg/m^3^ (FC1000). However, a slight decline in compressive strength is observed when the basalt fiber content increases beyond 0.6% up to 1.0%. Besides, all basalt fiber-reinforced foamed concrete samples exhibit higher compressive strengths compared to the foamed concrete samples without basalt fibers. In other words, the addition of basalt fiber effectively enhances the compressive strength of the foamed concrete. Furthermore, the cracking patterns also differ among samples with varying basalt fibers. Notably, basalt fibers effectively prevent the formation of transverse cracks, which are evident in the sample without basalt fibers. This observation highlights the substantial improvement in the toughness of foamed concrete due to the inclusion of basalt fibers. However, when the basalt fiber content exceeds 0.6%, a reduction in cohesion is observed, causing the surface layer of the foamed concrete to detach under compressive loading. This effect is particularly pronounced in the sample with a basalt fiber content of 1%.

The above analyses indicate that basalt fiber can effectively enhance the mechanical properties of foamed concrete, provided it is used within an optimal content range. The dynamic stress threshold, dynamic modulus, and damping ratio follow a similar trend, as shown in Fig. 6. As the basalt fiber content increases from 0% to 0.6%, the three parameters increase accordingly and reach their peak values. However, a gradual decline is observed as the basalt fiber content increases from 0.6% to 1.0%. All samples containing basalt fibers exhibit higher values in these parameters compared to those without basalt fibers, highlighting the improvement in the dynamic mechanical properties of the foamed concrete due to the presence of basalt fibers. Specifically, basalt fibers demonstrate a beneficial interaction with the cement matrix, effectively absorbing dynamic load energy and reinforcing the foamed concrete, thereby enhancing the dynamic stress threshold and dynamic modulus. However, an excessive content of basalt fibers results in an uneven distribution of pores within the foamed concrete, leading to a reduction in dynamic mechanical properties of samples. Therefore, determining the optimal fiber content is crucial for achieving the desired mechanical properties of the foamed concrete. Based on the test results and analysis, the optimal content of basalt fiber is found to be 0.6%. The main mechanical properties of foamed concrete at this optimal fiber content (BF0.6FC700, BF0.6FC1000) are summarized in Table 1.

**Table 1:** Main mechanical properties of the foamed concrete with a basalt fiber content of 0.6%

Density (kg/m^3^)	Compressive strength (MPa)	Dynamic stress threshold (MPa)	Dynamic modulus (MPa)	Damping ratio
700	3.39	1.02	1021.67	0.057
1000	6.80	1.45	1409.33	0.044

### Model construction and testing procedure

**Fig. 7:**
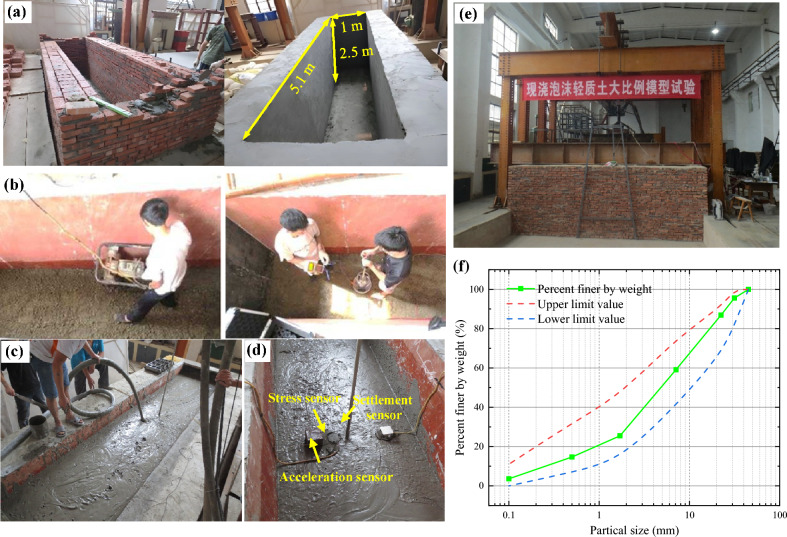
The construction process of the model: **a** building the brick wall; **b** foundation layer compaction and dynamic deformation modulus testing; **c** foamed concrete pouring; **d** testing sensors assignment; **e** the full-scale model; **f** the gradation curve of group B fillers

The model consisted of a foundation layer, foamed concrete layer, concrete sealing layer, and base plate layer, as shown in Fig. 3. The construction process of the model is illustrated in Fig. 7. The foundation layer, measuring 0.3 m in thickness, was filled with group B fillers. Following the soil test specification for railway engineering, a screening experiment was conducted to obtain the gradation curve of the group B fillers, as depicted in Fig. 7f. The non-uniform coefficient *C*_u_ and curve coefficient *C*_c_ were calculated as 28.24 and 2.24, respectively, indicating a relatively good grading of Group B fillers. The foundation layer was compacted using a small petrol-powered trench rammer, and the compaction effect was assessed through dynamic modulus testing. The compaction factor was confirmed to be 0.92, with modulus test results ranging from 25.4 to 31.7 MPa

**Fig. 8:**
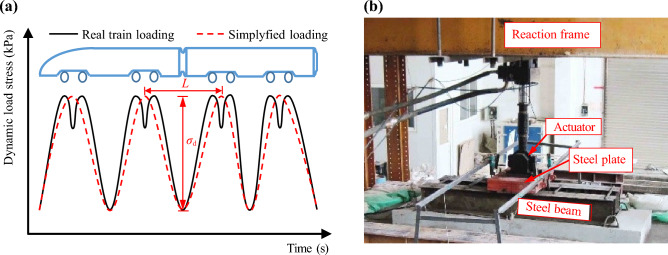
The loading procedure: **a** simulating loading curve; **b** the loading equipment

The foamed concrete layer consisted of the subgrade surface layer, with a thickness of 0.4 m, and the subgrade body, which was 1.75 m thick. The subgrade body was divided into four layers and filled sequentially. From the bottom to the top, the thicknesses of the layer were 0.45, 0.3, 0.5, and 0.5 m, respectively, all filled with BF0.6FC700. The surface layer of the subgrade bed was filled with BF0.6FC1000 in one continuous pour. After each layer was poured, the surface was covered with a geotextile film for 3 h of curing. Testing sensors—including dynamic acceleration sensors, dynamic stress sensors, vertical cumulative settlement sensors, and dynamic displacement sensors—were then installed, as shown in Fig. 3. Concurrently, dynamic deformation modulus (*E*_vd_) testing was performed. The results of the dynamic modulus tests for the subgrade body ranged from 171.55 to 196.73 MPa, while the results for the surface layer of the subgrade bed ranged from 237.18 to 266.48 MPa. The loading pulse exerted by a high-speed train on the subgrade surface is shown in Fig. 8. This loading pulse can be simplified as a single sine-wave load, as indicated in previous works [5, 33, 35–37]. Based on related research [36–38], the primary loading frequency *f* of the train-induced loads can be determined using the following equation:

1 $$ f = v/\left( {l \times 3.6} \right), $$

where *v* represents the velocity of the train in kilometers per hour (km/h), and *l* denotes the distance between adjacent bogies of the two vehicles (one in front and one behind) in meters (m), which is set to 7.5 m in this study. Using Eq. (1), the loading frequencies corresponding to various train speeds are determined and presented in Table 2. The loading amplitude *σ*_d_ is determined by the axle weight, which is set at 170 kN. It is important to note that the self-weight of both the track slab and rails is simulated by applying a static load via an actuator. Each loading frequency is subjected to loading 50,000 loading cycles, resulting in a total of 250,000 loading cycles. Detailed information about the loading procedure and data collection settings are provided in Table 2. For a visual representation of the loading equipment used in the research, refer to Fig. 8b.

**Table 2:** Loading parameters and data collection settings

Loading frequency (Hz)	Simulating speed (km/h)	Loading cycles	Data collection settings
6	162	50,000	Data were collected every 2,000 cycles when the loading cycles were ≤ 10,000. Beyond this point, the data collection intervals were extended to every 10,000 cycles
7	189	50,000	Collecting data every 10,000 cycles
9	243	50,000	Collecting data every 10,000 cycles
11	297	50,000	Collecting data every 10,000 cycles
13	351	50,000	Collecting data every 10,000 cycles

Note: Axle load is 17 t

## Test results and discussion

### Effects of the loading frequency on dynamic behavior

**Fig. 9:**
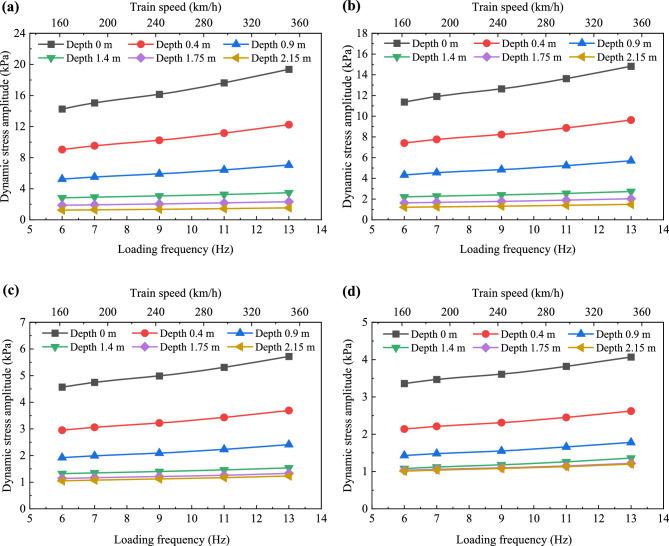
Variation in dynamic stress amplitudes with increasing loading frequency: **a** at the loading center, **b** 0.8 m from the loading center along the transverse direction, **c** 1.6 m from the loading center along the transverse direction, and **d** 2.4 m from the loading center along the transverse direction

**Fig. 10:**
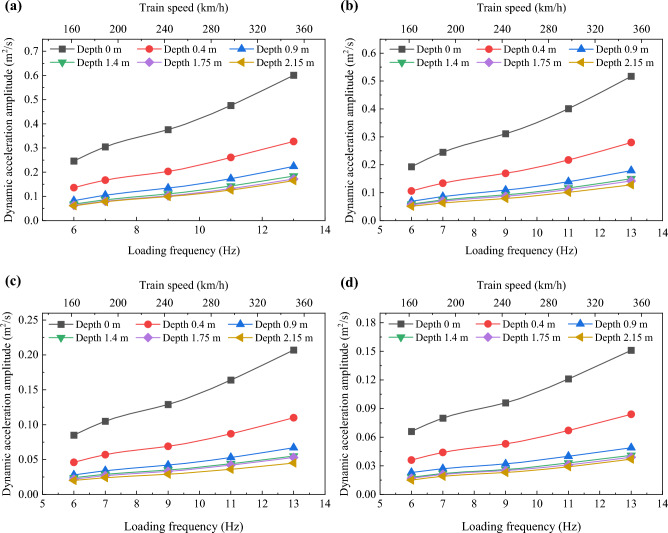
Variation in dynamic acceleration amplitudes with increasing loading frequency: **a** at the loading center, **b** 0.8 m from the loading center along the transverse direction, **c** 1.6 m from the loading center along the transverse direction, and **d** 2.4 m from the loading center along the transverse direction

Figures 9 and 10 illustrate the variations in dynamic stress amplitudes and dynamic acceleration amplitudes with respect to the loading frequency within the foamed concrete subgrade, respectively. It is evident that, regardless of depth, both dynamic stress amplitudes and dynamic acceleration amplitudes increase approximately linearly as the loading frequency rises, though the rates of increase remain small. Taking the dynamic stress amplitudes on the subgrade surface as an example, compared to the values at a loading frequency of 6 Hz, the average increases at 7, 9, 11, and 13 Hz are 4.25%, 10.35%, 18.35%, and 28.06%, respectively. These results suggest that, due to the good energy absorption properties of the foamed concrete and the continuous diffusion space in the subgrade, an increase in loading frequency (train speed) does not cause sudden changes in dynamic stress or acceleration within the subgrade. This characteristic is beneficial for the stability of high-speed railway (HSR) slab track systems.

As mentioned previously, while the peak values of dynamic stress and acceleration differ, their trends with loading frequency (train speed) are similar. To further explore the relationships between loading frequency and these two parameters, the Min–Max normalization method can be used to eliminate the influence of amplitude size, as shown in the following equation:

2 $$ x^{\prime } = \frac{{x_{i} - x_{{{\text{min}}}} }}{{x_{{{\text{max}}}} - x_{{{\text{min}}}} }}, $$

where $x^{\prime }$ is the normalized value; *x*_*i*_ represents the measured values of dynamic stress amplitudes (*σ*_*i*_) or dynamic acceleration amplitudes (*a*_*i*_) in a specific position; and the terms *x*_min_ and *x*_max_ correspond to the minimum and maximum values of the dynamic stress amplitude (*σ*_min_ and *σ*_max_) or the dynamic stress amplitude (*a*_min_ and *a*_max_), respectively.

**Fig. 11:**
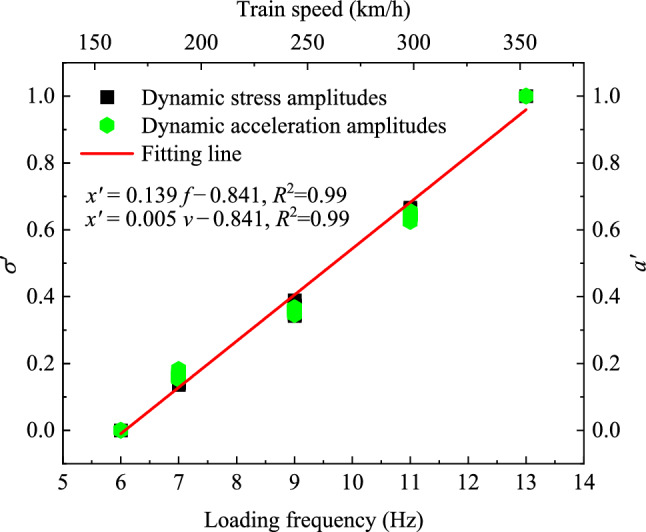
Min–Max normalization of dynamic stress amplitudes and dynamic acceleration amplitudes

Using Eq. (2), the normalized results of dynamic stress amplitudes and dynamic acceleration amplitudes at each measurement point are plotted in Fig. 11. It is evident that although the testing points are different, the normalized values of dynamic stress amplitudes and dynamic acceleration amplitudes are very close, and their variation trends with respect to loading frequency and train speed are similar. Specifically, the increase rates of $\sigma^{\prime }$ and $a^{\prime }$ with respect to loading frequency and train speed are 0.139 and 0.005, respectively. This indicates that the loading frequency has a limited influence on the dynamic response of foamed concrete subgrade. Furthermore, it is suggested that a simple linear function can be used to describe the relationship between these two indexes, loading frequency, and train speed, as shown below:

3 $$ \left. {\begin{array}{*{20}c} {\sigma^{\prime } \left( {{\text{or}} \;a^{\prime } } \right) = a_{1} v + b_{1} } \\ {\sigma^{\prime } \left( {{\text{or}} \;a^{\prime } } \right) = a_{2} f + b_{2} } \\ \end{array} } \right\}, $$

where *v* and *f* denote the train speed and loading frequency, respectively, while *a*_1_, *b*_1_, *a*_2_, and *b*_2_ serve as correlation coefficients. In this study, these coefficients were derived by fitting the normalized values, resulting in *a*_1_ = 0.139, *b*_1_ = 0.841, *a*_2_ = 0.005, and *b*_2_ = 0.841.

### Distribution of dynamic stress in the subgrade

**Fig. 12:**
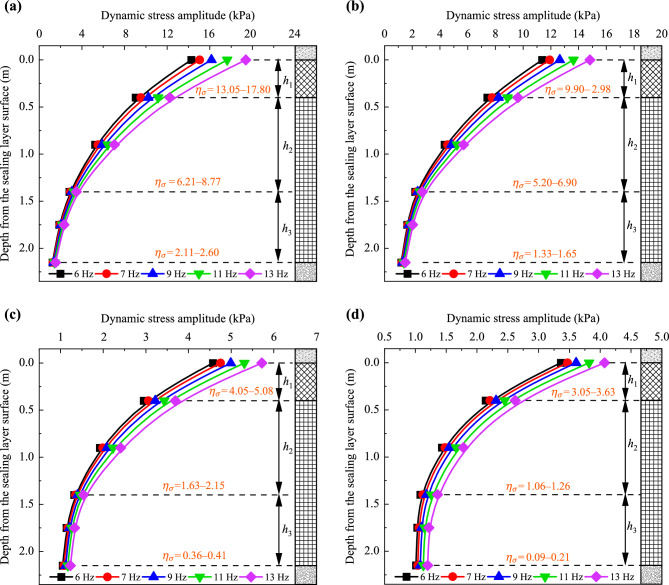
Dynamic stress amplitudes along the depth: **a** at the loading center, **b** 0.8 m from the loading center along the transverse direction, **c** 1.6 m from the loading center along the transverse direction, and **d** 2.4 m from the loading center along the transverse direction

The distribution of dynamic stress amplitudes along the depth is shown in Fig. 12. To further analyze the diffusion pattern of dynamic stress amplitudes along the depth, the coefficient *η* is proposed, which can be determined using the following equation:

4 $$ \eta_{\sigma } = \left| {\left. {\frac{{\sigma_{i1} - \sigma_{i2} }}{{h_{i1} - h_{i2} }}} \right|} \right. , $$

where *σ*_*i*1_ and *σ*_*i*2_ represent dynamic stress amplitudes at the lower depth and higher depths of the subgrade, respectively, while *h*_*i*1_ and *h*_*i*2_ denote the corresponding depths from the surface of the subgrade. The coefficient *η*_σ_ is used to evaluate the diffusion rate along the depth, and its values are presented in Fig. 12.

Figure 12 shows that the dynamic stress amplitudes at the subgrade surface, induced by simulated loading, range from 1.25 to 19.37 kPa. In contrast, field testing for the ballastless subgrade constructed with granular fillers shows values ranging from 8 to 30 kPa. This comparison indicates that a subgrade filled with foamed concrete can efficiently absorb dynamic energy and diffuse dynamic stress. Notably, the diffusion patterns of dynamic stress amplitudes along the depth remain consistent, regardless of train speed or testing position. Specifically, in the subgrade layer from the surface to a depth of 0.4 m, dynamic stress amplitudes decrease rapidly, with the diffusion coefficient *η*_*σ*_ reaching its highest value. This suggests that in this layer filled with BF0.6FC1000, vibration is most pronounced and is primarily absorbed and diffused in this zone. As the depth increases from 0.4 to 1.4 m, the dynamic stress amplitudes gradually decline. Beyond this, from 1.4 to 2.15 m, the dynamic stress amplitudes diminish slightly and tend to stabilize. Moreover, compared to the diffusion rate *η*_σ_ in the subgrade above the depth of 0.4 m, the values of *η*_*σ*_ decrease by approximately 47% to 65% between 0.4 and 1.4 m, and by 84% to 97% between 1.4 and 2.15 m. These findings suggest that the vibration is stronger in the foamed concrete subgrade above 0.4 m, with the influence of dynamic stress extending to approximately 1.4 m in depth.

**Fig. 13:**
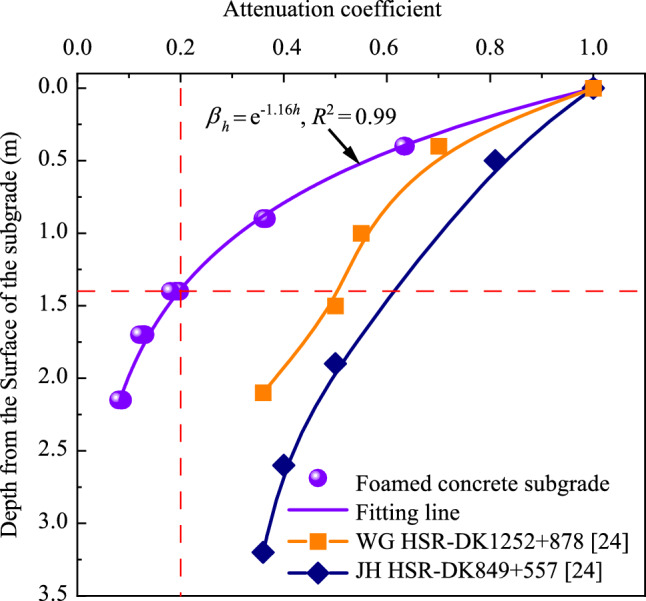
Attenuation patterns of dynamic stress along the depth beneath the loading center

Figure 13 illustrates the attenuation coefficients of dynamic stress amplitudes along depth, based on model testing data beneath the loading center and field measurements of the subgrade filled with granular fillers. The attenuation coefficient is defined as the ratio of the dynamic stress amplitude at any given depth to the value at the surface of the subgrade, under the same loading frequency. Compared with the field testing results [33] of Wuhan–Guangzhou high-speed railway (WG HSR) and Beijing–Shanghai high-speed railway (JH HSR) in China, it is evident that dynamic stress amplitudes attenuate more rapidly in the foamed concrete subgrade. Notably, in the foamed concrete subgrade at depths below 1.4 m, attenuation coefficients fall below 0.2. In contrast, for the subgrade of WG HSR or JH HSR, attenuation coefficients exceed 0.36 below the depth of 1.4 m. This indicates that the foamed concrete subgrade excels in absorbing and dissipating vibration energy, thus minimizing the depth of vibration impact. Furthermore, it is noteworthy that the relationship between the attenuation coefficient and the depth of the foamed concrete subgrade can be well approximated by an exponential equation, as depicted in Fig. 13.

**Fig. 14:**
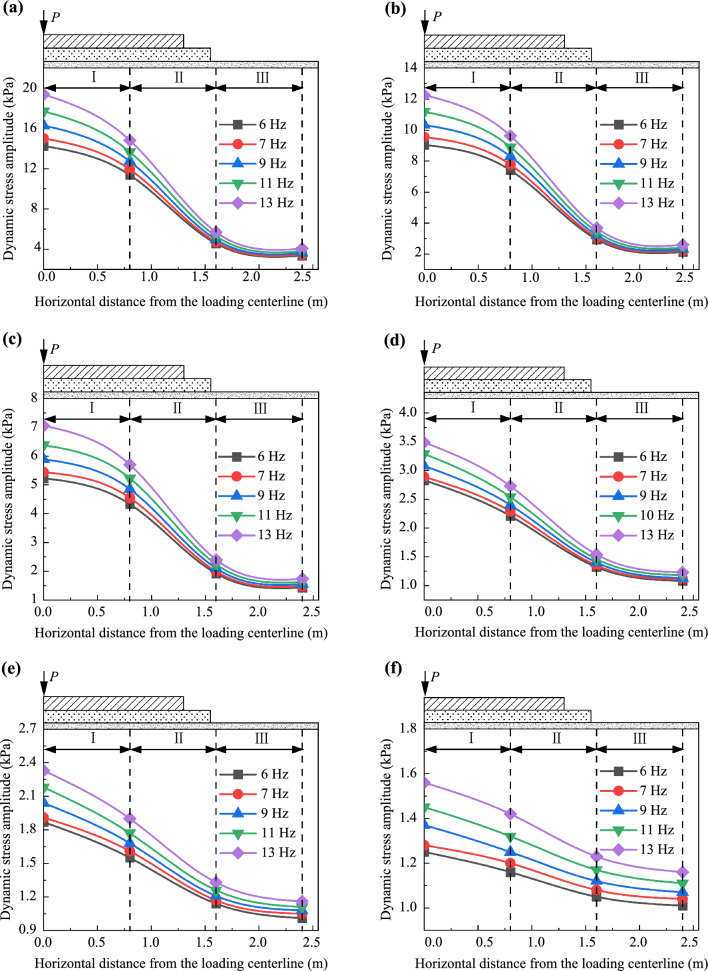
Dynamic stress amplitudes along the transverse direction of the subgrade **a** on the subgrade surface and at depths of **b** 0.40 m, **c** 0.90 m, **d** 1.40 m, **e** 1.75 m, and **f** 2.15 m

**Fig. 15:**
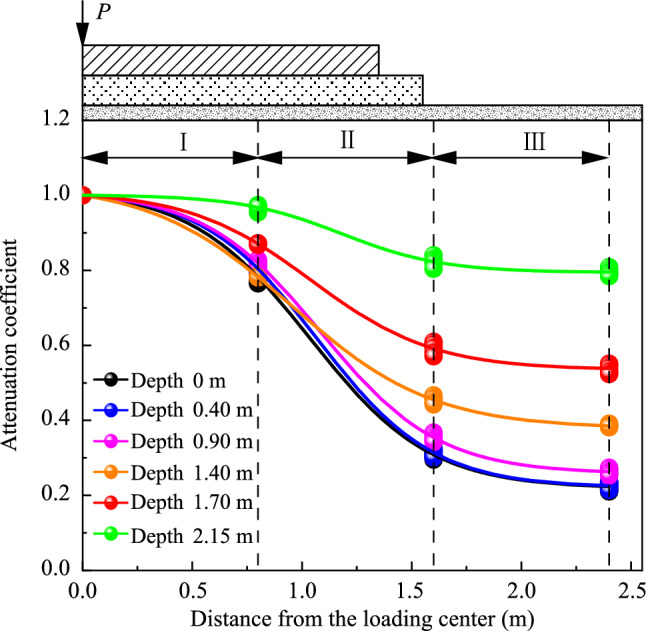
Attenuation patterns of dynamic stress along the transverse direction of the subgrade

Figures 14 and 15 depict the distribution and attenuation patterns of dynamic stress amplitudes along the lateral direction of the subgrade, respectively. The attenuation coefficient here refers to the ratio of the dynamic stress at any lateral position to the amplitude at the loading centerline, under the same loading frequency. Significantly, as shown in Figs. 14 and 15, the attenuation patterns of dynamic stress amplitudes along the transverse direction of the foamed concrete subgrade exhibit a similar trend, regardless of loading frequency or testing depth. Specifically, dynamic stress amplitudes decrease laterally across the subgrade. At a width of 0.8 m from the centerline, which corresponds to the middle of the base plate, attenuation coefficients all exceed 0.7 for loading frequencies ranging from 6 to 13 Hz. This indicates that vibrations are more intense in this region. At a width of 1.6 m, near the edge of the base plate, attenuation coefficients drop significantly, and the values at 1.6 m and 2.4 m from the centerline are quite similar. This suggests that vibration is absorbed within the subgrade up to a width of 1.6 m, particularly beneath the base plate. Additionally, the attenuation coefficients vary depending on the depth of the measurement within the subgrade, especially below a depth of 1.4 m. This demonstrates that the attenuation coefficient is influenced by the testing position within the subgrade.

**Fig. 16:**
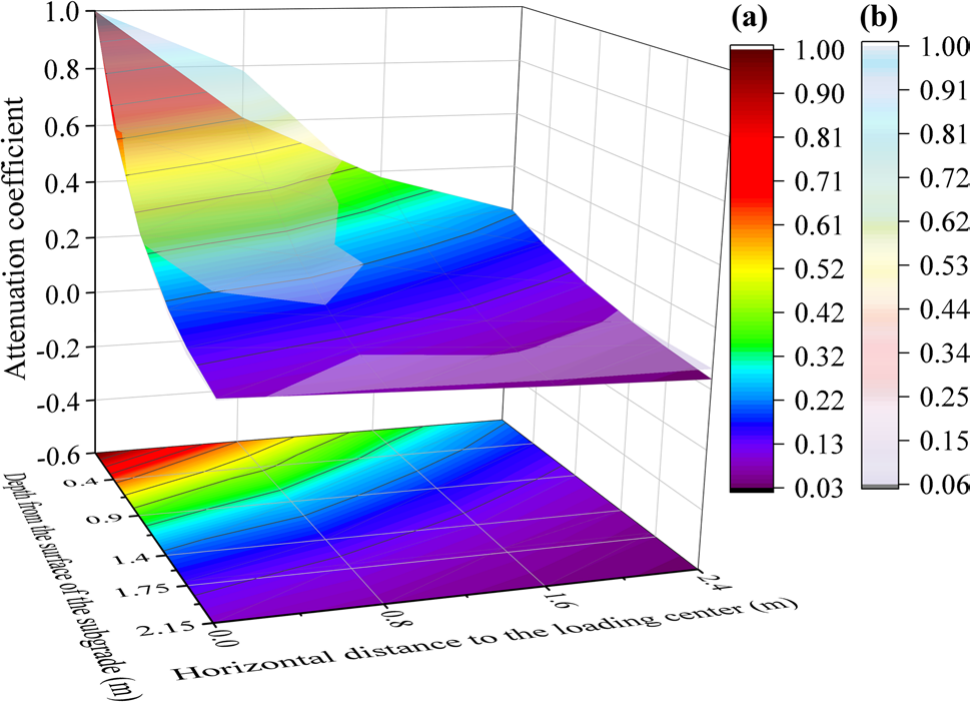
Attenuation coefficients of dynamic stress amplitudes in the subgrade: **a** model testing data; **b** calculated data

In this part, the attenuation coefficient *β* is defined as the ratio of dynamic stress amplitudes at any position within the subgrade to that at the loading center. The corresponding data are presented in Fig. 16. An empirical formula is proposed to calculate the values of the attenuation coefficient in the subgrade, as shown below:

5 $$\beta =\text{exp}\left[-\left(a\sqrt{{h}^{2}+{l}^{2}}+bh+cl\right)\right] , $$

where *h* and *l* represent the depth and width of the testing position in the subgrade, respectively, while *a*, *b*, and *c* are correction coefficients. All these factors are dimensionless. By fitting the model testing data, the values of *a*, *b*, and *c* are 0.365, 0.779, and 0.234, respectively, with an *R*-squared value of 0.96 for Eq. (5).

It is evident that the attenuation coefficients of dynamic stress vary with the position within the subgrade, implying that *a*, *b*, and *c* can also function as correction factors for these values at different locations in the subgrade. Furthermore, as a typical layered anisotropic geotechnical structure, the thickness of each layer and the damping ratio of the materials significantly influence vibration diffusion. Therefore, based on the test data and inverse analysis, the following equations are proposed to calculate these three coefficients:

6a $$ a = 3\sum {h_{i} \lambda_{i}} ,$$

6b $$ b = \overline{{\left( {\frac{{\lambda_{i} }}{{\lambda_{1} }}} \right)}} ,$$

6c $$ c = \overline{{\left( {\frac{{\lambda_{i} }}{{\lambda_{1} }}} \right)}} /3, $$

where *h*_*i*_ and *λ*_*i*_ represent the thickness of the different layers filled with various densities of foamed concrete and the damp ratio of the foamed concrete, respectively; *λ*_1_ is the damp ratio of the foamed concrete used to fill the surface layer of the subgrade. It should be noted that all parameters here are dimensionless.

In this model, the surface layer of the subgrade with a thickness of 0.4 m was filled with BF0.6FC1000, and below the surface layer of the subgrade, a layer with a thickness of 1.75 m was filled with BF0.6FC700. As shown in Table 1 and Fig. 3, the damping ratios of BF0.6FC1000 and BF0.6FC700 are 0.044 and 0.057, respectively. The theoretical calculation results are plotted out in Table 3, and interestingly the proportional errors between the model testing data fitting values and theoretical calculation values for the three coefficients are 3.6%, 9.0%, and 9.8%. This indicates that the theoretically calculated correction coefficients are reliable. The distribution curved surface of the attenuation coefficient *β* obtained from the theoretical calculation is shown in Fig. 16. Thus, Eq. (5) is proposed to assess the diffusion pattern of dynamic stress in the foamed concrete subgrade.

**Table 3:** Values of correction coefficients

Coefficient	Values from model testing data fitting	Values from theoretical calculation	Difference (%)
*a*	0.365	0.352	3.6
*b*	0.779	0.772	9.0
*c*	0.234	0.257	9.8

### Distribution of dynamic acceleration in the subgrade

**Fig. 17:**
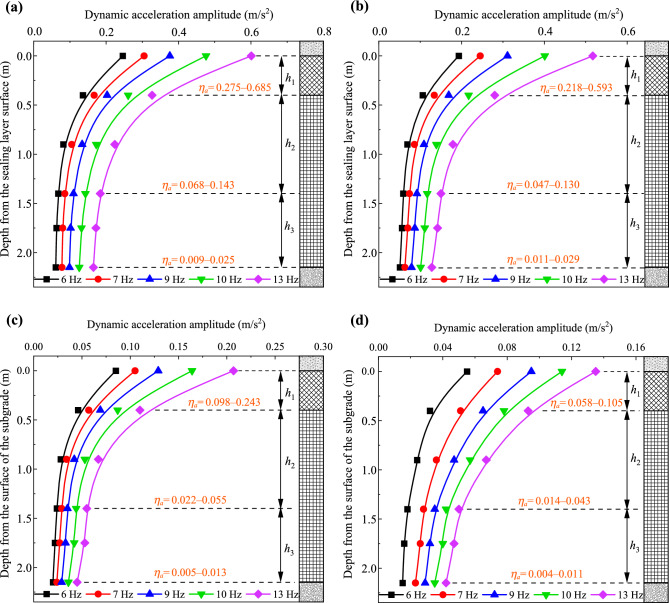
Dynamic acceleration amplitudes along the depth: **a** at the loading center, **b** 0.8 m from the loading center along the transverse direction, **c** 1.6 m from the loading center along the transverse direction, and **d** 2.4 m from the loading center along the transverse direction

**Fig. 18:**
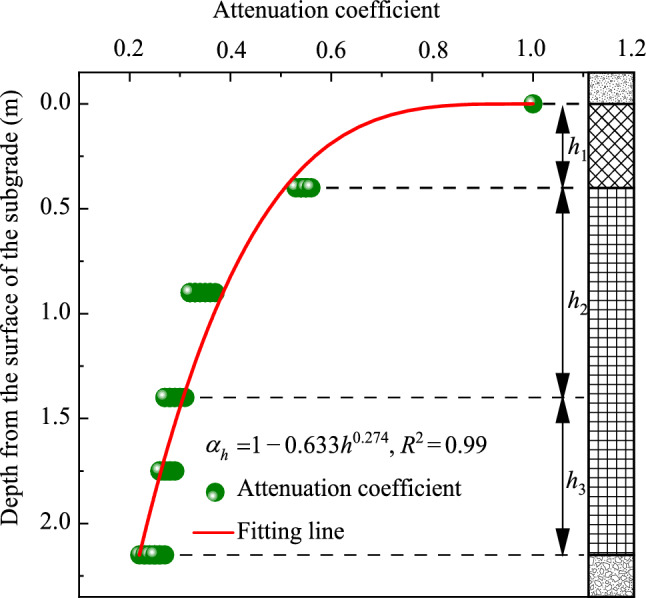
Attenuation coefficients of dynamic acceleration along the depth

Figure 17 illustrates the dynamic acceleration amplitude variations within the subgrade as a function of depth, while Fig. 18 depicts the attenuation coefficient of dynamic acceleration for depth. It is worth emphasizing that the attenuation coefficient, denoted as *α*_*h*_, signifies the ratio of dynamic acceleration amplitudes at any depth to those at the surface of the subgrade. Furthermore, the coefficient *η*_*a*_ characterizes the diffusion rate of dynamic acceleration amplitudes along the depth of the subgrade, as determined by Eq. (7):

7 $$ \eta_{a} = \left| {\left. {\frac{{a_{i1} - a_{i2} }}{{h_{i1} - h_{i2} }}} \right|} \right. ,$$

where *a*_*i*1_ and *a*_*i*2_ represent dynamic stress amplitudes at lower and higher depths of the subgrade, respectively, while *h*_*i*1_ and *h*_*i*2_ denote the respective depths relative to the surface of the subgrade.

Figure 17 reveals that dynamic acceleration amplitudes at the subgrade surface under various train speeds range from 0.06 to 0.60 m/s^2^. In contrast, for the subgrade filled with granular materials, these values range from 7.80 to 16.89 m/s^2^, based on numerical analysis [39]. This indicates that the vibration inside the foamed concrete subgrade is significantly weaker compared to that in a subgrade filled with granule materials, which is advantageous for ensuring the smooth operation of high-speed railways. As shown in Fig. 17, dynamic acceleration amplitudes decrease rapidly from the surface to a depth of 0.4 m in the subgrade. From the depth of 0.4 m to 1.4 m in the subgrade, these values decline more gradually, while between 1.4 m and 2.15 m, they exhibit a minor reduction and tend to stabilize. Additionally, the diffusion rate *η*_*a*_ from the surface to 0.4 m in the subgrade is approximately 2.5–4.5 times larger than that from 0.4 to 1.4 m, and 10–25 times larger than that from 0.4 to 1.4 m.

Interestingly, as shown in Fig. 18, although the loading frequency and lateral position of the test points are different, all distribution curves of the attenuation coefficient along the depth exhibit show a similar trend, and the attenuation coefficient values at the same depth are very close. Specifically, under different loading frequencies, dynamic acceleration amplitudes at depths of 0.4, 1.4, and 2.15 m are approximately 0.53 to 0.56, 0.27 to 0.31, and 0.22 to 0.27 times those at the surface of the subgrade, respectively. These analyses suggest that vibration induced by loading is significantly stronger within the subgrade above a depth of 0.4 m, where the diffusion is also the most rapid. Benefiting from the outstanding energy absorption properties and diffusion effects of the foamed concrete subgrade, vibration in the subgrade bottom layer, situated below a depth of 1.4 m, is notably weaker. This also implies that the effective influence depth of the dynamic acceleration is approximately 1.4 m. Furthermore, the diffusion patterns of dynamic acceleration amplitudes along the depth exhibit a consistent trend regardless of the width of the testing position within the subgrade or the loading frequency. Based on the model testing data, the attenuation coefficient along the depth (*α*_*h*_) can be calculated using the following equation:

8 $$ \alpha_{h} = 1 - A_{1} h^{{B_{1} }} ,$$

where *h* is the depth within the subgrade, and *A*_1_ and *B*_1_ are correction coefficients with values of 0.633 and 0.274, respectively.

**Fig. 19:**
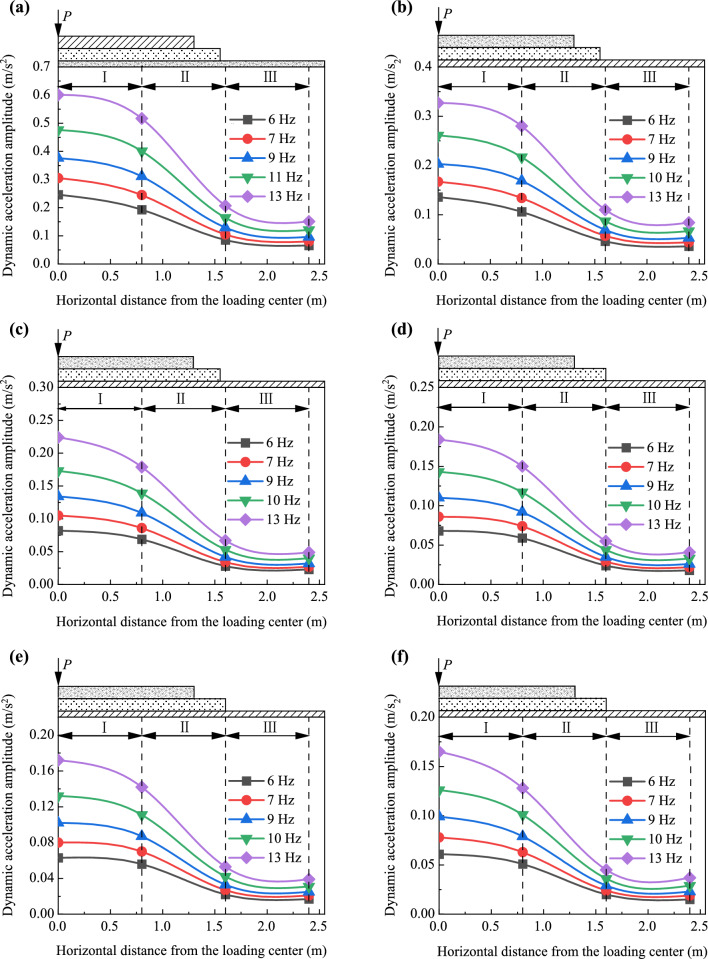
Dynamic acceleration amplitudes along the transverse direction of the subgrade **a** on the subgrade surface and at depths of **b** 0.40 m **c** 0.90 m, **d** 1.40 m, **e** 1.75 m, and **f** 2.15 m

**Fig. 20:**
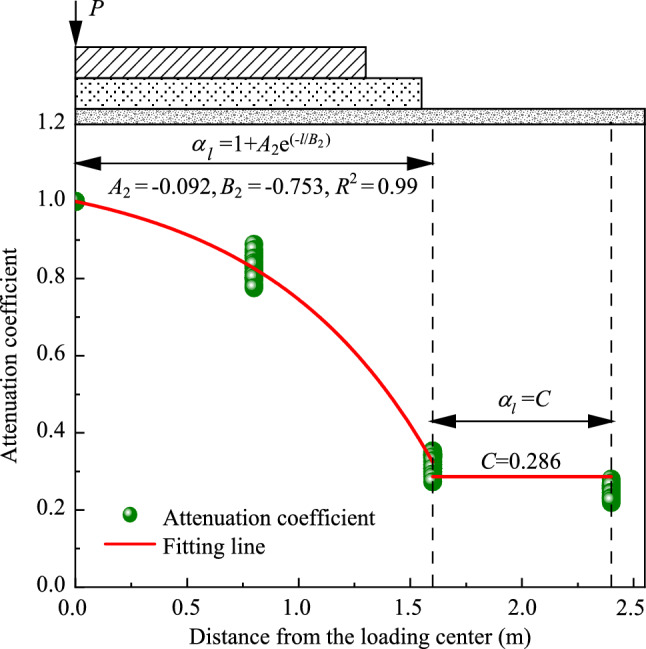
Attenuation coefficients of dynamic acceleration along the transverse direction of the subgrade

Figures 19 and 20 depict the distribution and attenuation patterns of dynamic acceleration amplitudes along the transverse direction of the foamed concrete subgrade, respectively. The attenuation coefficient along the width (*α*_*l*_) in this part signifies the ratio of the dynamic acceleration amplitude at any transverse position to that at the loading center, under the same loading frequency and at the same depth. Notably, Fig. 19 demonstrates that diffusion patterns of dynamic acceleration amplitudes along the transverse direction remain consistent, regardless of the depth of the testing position or the loading frequency. Specifically, dynamic acceleration amplitudes decrease gradually within the subgrade from the loading center to a width of 0.8 m. From the width of 0.8 m to 1.6 m, the values decrease more rapidly, and from 1.6 m to 2.4 m, the amplitudes decrease smoothly and tend to stabilize.

This diffusion pattern is more comprehensively illustrated in Fig. 20. The values of the attenuation coefficient along the transverse direction (*α*_*l*_) at a width of 0.8 m from the loading centerline of the subgrade range from 0.78 to 0.89, while the values at widths of 1.6 m and 2.4 m in the subgrade range from 0.35 to 0.27 and 0.28 to 0.22, respectively. This observation further suggests that the vibration is considerably stronger within a width of 0.8 m, corresponding to the central region of the base plate. The effective diffusion distance along the transverse direction of the subgrade is approximately 1.6 m, which is near the edge of the base plate. Through calculations, the diffusion pattern of dynamic acceleration amplitudes along the transverse direction of the foamed concrete subgrade can be described by a piecewise function, as shown below:

9 $$ a_{l} = \left\{ {\begin{array}{*{20}l} {1 + A_{2} {\text{e}}^{{ - l/B_{2} }} } \hfill & {{\text{for }}\;0{\text{ m}} < l \le 1.6{\text{ m}}} \hfill \\ C \hfill & { {\text{for }}l > 1.6{\text{ m}}} \hfill \\ \end{array} } \right. , $$

where *l* presents the distance from the loading center line, *A*_2_ and *B*_2_ are correction coefficients, and *C* is a constant. In this study, the values of *A*_2_, *B*_2_, and *C* obtained by fitting the model testing data are −0.092, −0.753, and 0.286, respectively.

### Dynamic displacement and cumulative settlement

**Fig. 21:**
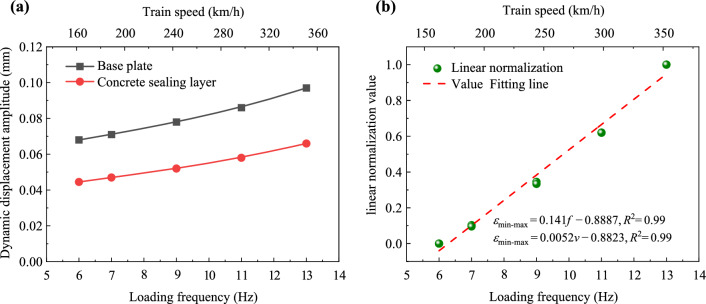
Dynamic displacement amplitudes: **a** variation of the values with increasing loading frequencies; **b** linear normalization

The variations in dynamic displacement amplitudes within the subgrade as a function of loading frequency are plotted in Fig. 21. The linear normalization values are also obtained using Eq. (2). It is evident that dynamic displacement amplitudes increase gradually and only slightly with the rise in loading frequency (train speed). That indicates that when the train speed increases from 162 to 351 km/h, the impact on the vibration of the foamed concrete subgrade is minimal. Moreover, compared with the dynamic displacement amplitudes at the surface of the base plate, the values at the surface of the concrete sealing layer decrease by approximately 32% to 35%. This demonstrates that the foamed concrete subgrade effectively absorbs and diffuses dynamic pulse energy, thereby enhancing the stability of the track structure’s dynamic performance during operation. Using the Min–Max normalization method to process dynamic displacement data, it becomes clear that the relationship between loading frequency and *ε*_min-max_ values, as well as the relationship between these values and train speed, can both be described by a unary linear function.

**Fig. 22:**
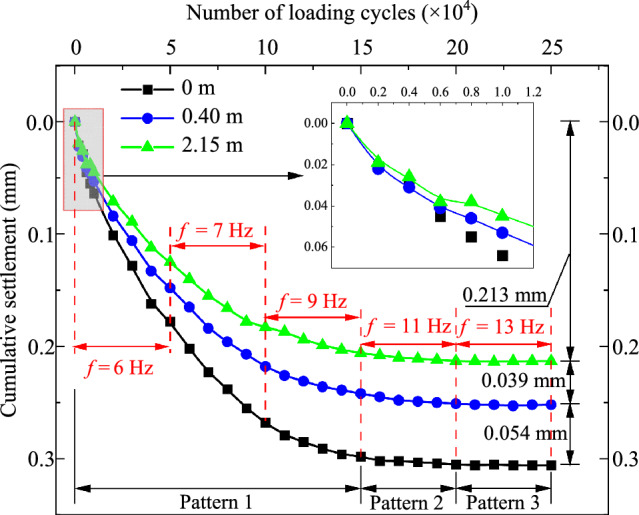
Cumulative settlement with the number of loading cycles

Figure 22 shows the evolution of cumulative settlement of the subgrade at different depths as a function of the number of loading cycles. Obviously, the cumulative settlement curves follow a similar trend across all testing positions as the number of loading cycles increases. Specifically, in the early stage of loading, before approximately 0.8 × 10^4^cycles, the cumulative settlement at each depth grows with fluctuations. After this initial stage, the cumulative settlement increases rapidly until the number of loading cycles reaches 15 × 10^4^. At this time, the cumulative settlement at the surface, 0.4 m depth, and 2.15 m depth reaches 0.298, 0.242, and 0.206 mm, respectively, each exceeding 96% of their final values. Figure 22 also illustrates that the cumulative settlement curve can be divided into three stages. Stage 1 occurs from the beginning to 10 × 10^4^ cycles, during which most of the cumulative settlement takes place. Stage 2 spans from 15 × 10^4^ to 20 × 10^4^ cycles, where the cumulative settlements at the three testing positions increase slightly to 0.305, 0.251, and 0.213 mm, respectively. Stage 3, occurring between 20 × 10^4^ and 25 × 10^4^ cycles, marks the stabilization of cumulative settlements. At the conclusion of the loading process, the final values at the surface, 0.4 m depth, and 2.15 m depth of the foamed concrete subgrade are 0.306, 0.252, and 0.213 mm, respectively.

Furthermore, the compressive deformations of different layers of the subgrade, calculated from the testing data, are shown in Fig. 22. This figure indicates that the compressive deformation of the foundation is 0.213 mm, which accounts for approximately 69.7% of the final cumulative settlement. Meanwhile, the deformations for the foamed concrete layers filled with BF0.6FC700 and BF0.6FC1000 are 0.039 mm and 0.054 mm, respectively. This suggests that under the long-term loading cycles, the deformation of the foamed concrete is minimal, which is advantageous for controlling subgrade settlement. Moreover, it is evident that the number of loading cycles significantly impacts cumulative settlement and compressive deformation, whereas these values are less sensitive to loading frequency. In other words, the train speed has a minimal effect on both the cumulative settlement and the compressive deformation of the foamed concrete.

## Conclusions

In this study, a series of uniaxial compressive strength tests and dynamic triaxial tests were performed. A full-scale test track with simulated train moving loads has been set up, and five different frequency dynamic loading tests were conducted to explore the dynamic performance and cumulative settlement of the foamed concrete subgrade in high-speed railways. Based on the model testing, the following conclusions can be drawn:

(1) When the densities of foamed concrete are 700 and 1000 kg/m^3^, the mechanical properties of the material can be effectively enhanced by incorporating 0.6% basalt fibers with a diameter of 13 μm and a length of 6 mm.

(2) Across train speeds ranging from 162 to 351 km/h, dynamic stress amplitudes, and dynamic acceleration amplitudes within the foamed concrete subgrade, and dynamic displacement amplitudes at the surface of the track structure exhibit only marginal increases with train velocity. Variations in train speed have a limited impact on the vibration diffusion patterns inside the foamed concrete subgrade.

(3) In the foamed concrete subgrade, dynamic stress, and dynamic acceleration responses are strongest within the top 0.4 m of depth and up to a width of 0.8 m. These response indexes gradually diffuse along the width and depth directions of the subgrade, stabilizing within the subgrade below a depth of 1.4 m and beyond a width of 1.6 m.

(4) Dynamic stress and acceleration demonstrate nonlinear diffusion within the foamed concrete subgrade. Based on model testing results, a multivariate natural exponential function is proposed to describe the attenuation pattern of dynamic stress inside the foamed concrete subgrade. Additionally, the attenuation of dynamic acceleration along the depth and width of the subgrade can be described by a power function and a natural exponential function, respectively.

(5) The cumulative settlement of the subgrade increases rapidly with the number of loading cycles, reaching a peak around 15 × 10^4^ cycles. Beyond this, in the settlement experiences minor fluctuations, ultimately stabilizing after 25 × 10^4^ cycles. The final cumulative settlement of the subgrade is measured at 0.306 mm, with compressive deformations of the foamed concrete layers induced by simulated train loading remaining below 0.1 mm. The ultimate cumulative settlement is primarily attributed to foundation compression.

The full-scale experimental testing provides valuable insights into the dynamic behavior of the foamed concrete subgrades. These findings are beneficial for the design and maintenance of slab track high-speed subgrades filled with foamed concrete. Future numerical simulations could further analyze the dynamic behavior of the foamed concrete subgrades and optimize the empirical formula proposed in this study.
